# Ice Templated PEG–Alginate Double-Network Cryogels with Tunable Mechanics and Degradation for Soft Tissue Engineering

**DOI:** 10.3390/gels12060533

**Published:** 2026-06-13

**Authors:** Kaixiang Zhang, Michael Patrick Seitz, Matthew Pinto, William Ofori-Atta Eghan, Era Jain

**Affiliations:** 1 Department of Biomedical and Chemical Engineering, Syracuse University, Syracuse, NY 13244, USA; kzhang57@syr.edu (K.Z.); mseitz@syr.edu (M.P.S.); mpinto1@terpmail.umd.edu (M.P.); woforiat@syr.edu (W.O.-A.E.); 2 Bioinspired Syracuse: Institute for Material and Living System, Syracuse University, Syracuse, NY 13244, USA

**Keywords:** double network, cryogels, cartilage tissue engineering, hydrogels, macroporous scaffolds, stem cell-macrophage crosstalk

## Abstract

Scaffolds designed for mechanically demanding soft tissue engineering applications should integrate mechanical support, efficient mass transfer, and good cellular compatibility. This work presents a one-pot method based on “radical-free click chemistry + carbodiimide coupling” to produce a double-network (DN) PEG–alginate cryogel. The PEG network is formed by a Michael addition reaction between thiol-based crosslinker and 8-arm PEG-acrylate. The second network is covalently crosslinked through EDC/NHS-mediated coupling of carboxyl groups in alginate and adipic acid dihydrazide (AAD). The subsequent freezing and gelation of the gel precursor at sub-zero temperatures results in an ice templated cryogel with an interconnected macroporous network. These cryogels demonstrate high elasticity, compressive modulus and rapid swelling equilibrium in aqueous environments, as well as controlled degradation under physiological conditions. Compared to the classical Ca^2+^ ion crosslinking systems, the covalent linking of the alginate in the double-network cryogel shows advantages in mechanical and structural stability. In addition, it is cell-compatible and allows culture of mesenchymal stem cells (MSCs) with homogeneous infiltration. Furthermore, the double-network cryogels supports chondrogenic differentiation of MSCs upon treatment with chondrogenic media or macrophage-conditioned media for a short period of time. These results indicate that crosslinking chemistry and polymer composition can be used to modulate the balance between mechanical performance and degradation behavior, while maintaining cytocompatibility and an interconnected macroporous network, thereby providing a scaffold design strategy for applications that require coordinated mechanical support and mass transfer, such as cartilage-related tissue engineering.

## 1. Introduction

Tissue engineering and wound healing strategies aim to promote tissue repair by synergistically harnessing the body’s intrinsic healing capacity together with implanted biomaterials and bioactive factors [1,2,3]. Biocompatible scaffolds are essential in these strategies, offering temporary structural support, promoting cell infiltration, and enabling the controlled release of therapeutic signals throughout regeneration [4]. As a result, biodegradable porous scaffolds, with hydrogels at the forefront, have become a major area of focus in soft tissue engineering [5,6].

Hydrogels have been extensively regarded as promising materials due to their tissue-like properties, high hydration level, and excellent biocompatibility [7]. Nevertheless, traditional hydrogels remain hindered by insufficient mechanical strength and toughness, which reduces their suitability for mechanically demanding applications [8,9]. To address these limitations, double-network (DN) hydrogels have arisen as a promising approach for enhancing mechanical toughness and robustness [10,11]. Despite these advantages, many DN hydrogels lack key features required for translational applications, including cytocompatible fabrication, controlled biodegradability, and interconnected porosity necessary for efficient mass transport and cell infiltration [11,12,13].

Cryogels are a unique class of hydrogel synthesized at subzero temperatures to produce macroporous, highly compressible, and convective mass-transporting structures with high elasticity [14,15,16]. Cryogels are uniquely favorable for tissue engineering, because they allow the diffusion of nutrients, support cell seeding and infiltration at high cell densities, and are mechanically resilient [17,18]. During cryogelation, water crystallizes into ice, which functions as a porogen, whereby the gel precursors are enriched in the unfrozen microphase and undergo crosslinking [19,20]. 

Alginate, a naturally occurring polysaccharide, has found wide application in biomedical fields owing to its biocompatibility and gelling capability in presence of divalent cations [21,22]. Although ionic crosslinking under cryogenic conditions is a biocompatible approach for fabricating degradable alginate cryogels, these systems generally suffer from poor mechanical strength and limited reproducibility during cryogelation [23,24,25]. While alginate cryogels have been successfully synthesized, many rely on free-radical polymerization, or click-based crosslinking, which can adversely affect biodegradability [26,27]. To enable radical-based or click-based covalent crosslinking at sub-zero temperatures, alginate is often pre-functionalized or oxidized; however, this strategy offers limited tunability of mechanical properties and degradability, as crosslinking density is largely dictated by the fixed degree of pre-functionalization. Moreover, extensive pre-functionalization can also affect biocompatibility [28]. In contrast, alginate hydrogels can be fabricated using adipic acid dihydrazide (AAD), a biocompatible crosslinker that covalently links alginate carboxyl groups via carbodiimide chemistry. By avoiding alginate pre-functionalization, this approach enables the construction of a stable network and offers increased flexibility for tuning crosslinking density and the resulting material properties [29,30].

Polyethylene glycol (PEG) has become a commonly used polymer in tissue engineering, largely due to its tunable physicochemical properties and biological inertness [31]. PEG cryogels are generally prepared by free-radical polymerization, producing non-degradable networks [29,30,32,33]. PEG hydrogels or cryogels prepared through Michael-type addition reactions between thiol and acrylate moieties provide hydrolytically degradable networks [25]. By selecting cross-linkers with different chemical structures, the degradation rate for these hydrogels and cryogels can be finely tuned [23,34,35].

In this study, we introduce a one-pot, radical-free dual-crosslinking strategy that integrates click chemistry and carbodiimide coupling to fabricate macroporous, biodegradable PEG–alginate double-network (DN) cryogels [25]. The alginate network is formed through covalent carbodiimide-mediated coupling of carboxyl groups to AAD. The PEG network is established via Michael-addition reaction between thiol-based crosslinkers and multi-arm PEG acrylates, yielding an elastic and highly mechanically tunable network. The integration of these two networks synergistically enhances mechanical strength and provides long-term structural stability. By combining relatively stable hydrazide crosslinks with hydrolytically labile thioether ester crosslinks, this platform enables formulation-dependent modulation of degradation behavior while maintaining a mechanically robust double-network structure. The results suggest that PEG content and dithiol crosslinker chemistry can be used to adjust the balance among mechanical properties, swelling behavior, pore architecture, and degradation kinetics. The resulting DN cryogels exhibit interconnected macroporosity, rapid swelling behavior, high cytocompatibility and early chondrogenic differentiation. Furthermore, modulation of PEG content enabled systematic tuning of degradation rates. Collectively, this platform represents a flexible scaffold strategy in which compressive properties, swelling behavior, and degradation-associated mass loss can be modulated through polymer composition and crosslinker chemistry. These features suggest potential relevance for mechanically demanding soft tissue engineering applications, including cartilage-related scaffold design.

## 2. Results and Discussion

### 2.1. Synthesis of Cryogels

We first, optimized the synthesis conditions for the PEG–alginate double-network (DN) cryogels. Based on our previous work, a freezing temperature of −20 °C was selected as optimal for DN cryogel formation [25]. During preparation of the crosslinker mixture containing AAD, we observed that precooling the solution on ice prior to freezing led to visible AAD precipitation. Cryogels synthesized using these mixtures either failed to form or were highly fragile, indicating variability in crosslinking under frozen conditions and poor reproducibility. This behavior is consistent with the limited solubility of AAD in cold aqueous solutions, where cooling of near-saturated solutions promotes precipitation.

To address this issue, we evaluated multiple AAD concentrations. Although reducing the precooling time decreased the likelihood of precipitation, it compromised reproducibility across batches. We therefore pivoted to adjusting AAD content and tested formulations containing 0.8 M, 0.65 M, and 0.5 M of AAD. At 0.5 M AAD, precipitation was no longer observed. DN cryogels synthesized under these conditions were highly reproducible and exhibited robust mechanical integrity upon manual handling, indicating effective crosslinking and stable network formation.

We also evaluated alginates from two different commercial sources. Manugel GMB alginate (M/G ratio 0.6; MW 124 × 10^3^–10^5^ g mol^−1^; viscosity 180 cps) produced highly viscous solutions at elevated concentrations, hindering uniform mixing of crosslinkers and reliable cryogel formation. In contrast, Protanal^®^ LF 10/60 alginate (M/G ratio 0.6; MW 81 × 10^3^–31 × 10^4^ g mol^−1^; viscosity 55 cps) yielded manageable high-concentration solutions and enabled consistent DN cryogel synthesis. Accordingly, all subsequent DN cryogels were fabricated using Protanal LF 10/60.

The PEG network was formed through Michael-addition click crosslinking between the dithiol crosslinker and the multi-arm PEG, enhancing the mechanical stability of the DN cryogels. We varied the concentration of multiarm-PEG acrylate from 5 to 20% with the DN cryogels to obtain 20% PEG–alginate, 10% PEG–alginate, and 5% PEG–alginate DN cryogels. Additionally, we evaluated the effect of two different dithiol crosslinkers, DTT and EGBMA on the formation of PEG network and DN cryogels, due to the similarity in structure and molecular weight but different susceptibility to hydrolytic degradation [25,35,36,37,38,39]. 

Despite the changing concentration of PEG and the dithiol crosslinker in the DN cryogels, the carbodiimide-mediated coupling chemistry involving EDC, NHS, and AAD resulted in covalent crosslinking within the alginate network, whereby activation of the carboxyl group by EDC produces an O-acylisourea intermediate, which subsequently reacts with the hydrazide of AAD to yield a stable substituted hydrazide (acylhydrazide) bond. Since AAD has two hydrazides, it leads to crosslinking between alginate chains [29,30,34].

This design differs from our previously reported PEG–alginate hybrid DN cryogels, in which the alginate phase was ionically crosslinked by Ca^2+^. Here, the alginate network is covalently crosslinked by AAD through carbodiimide chemistry. This change is important because the alginate network no longer has reversible ionic crosslinks. As shown further, covalently crosslinking improved compressive modulus compared with the earlier ionically crosslinked system suggesting that covalent alginate crosslinking contributes to the mechanical performance of the DN cryogels.

### 2.2. Microstructure of PEG–Alginate DN Cryogels

**Figure 1:**
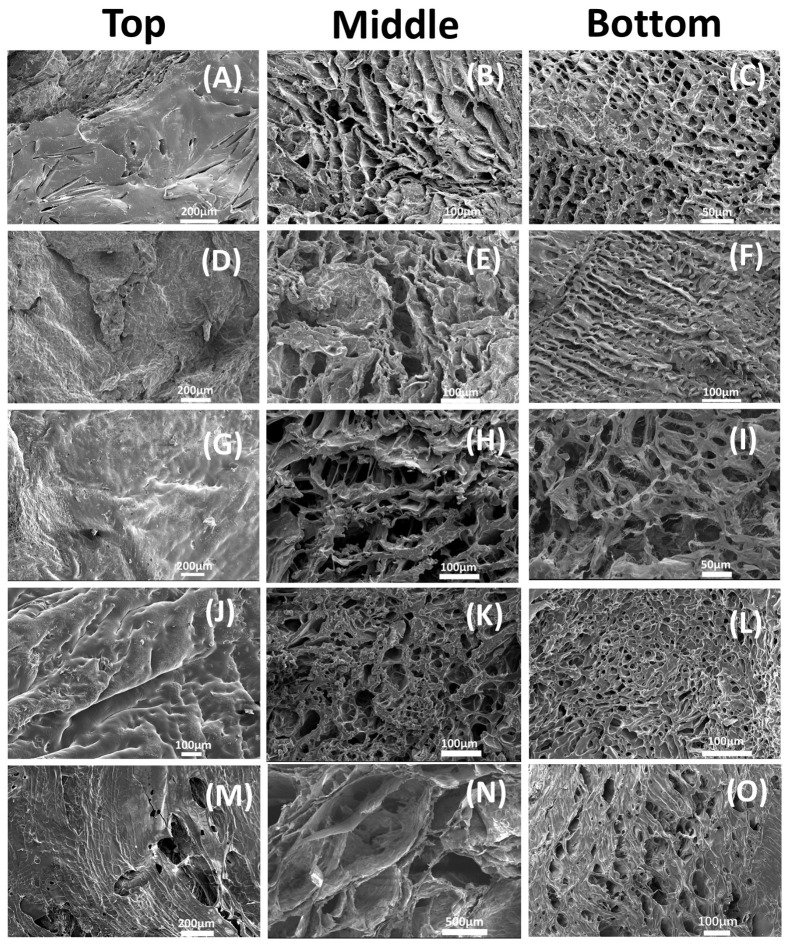
SEM images of PEG–alginate double-network (DN) cryogels. Cross-sectional regions from top, middle, and bottom were examined for each cryogel formulation. (**A**–**C**) 20% PEG–alginate-DTT DN cryogels; (**D**–**F**) 10% PEG–alginate-DTT DN cryogels; (**G**–**I**) 5% PEG–alginate-DTT DN cryogels; (**J**–**L**) 20% PEG–alginate-EGBMA DN cryogels; and (**M**–**O**) alginate–AAD single-network (SN) cryogels.

**Figure 2:**
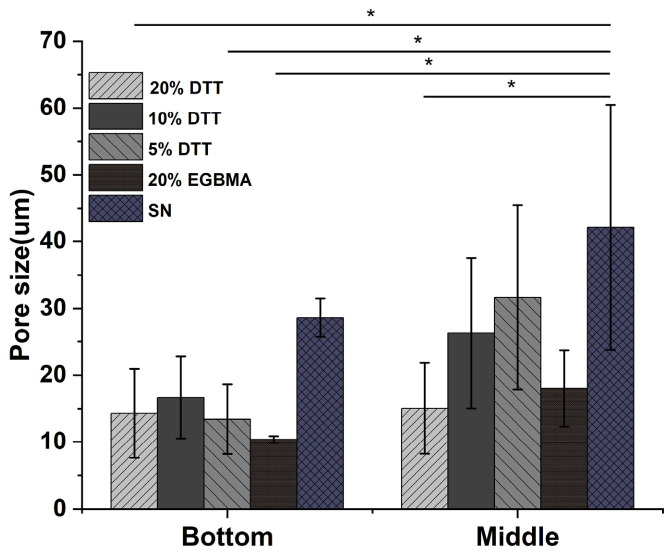
Distributions of pore size in the middle and bottom cross-sectional regions of PEG–alginate double-network (DN) cryogels and alginate single-network (SN) cryogels. Differences in pore size among the cryogel groups were statistically assessed through one-way ANOVA followed by Tukey’s post hoc test; * indicates *p* < 0.05 (*n* = 6).

Scanning electron microscopy (SEM) imaging revealed that the DN PEG–alginate cryogels possess a highly interconnected macroporous structure characteristic of cryogelation (Figure 1). The pore architecture displayed elongated, anisotropic, and irregularly shaped pores distributed throughout the scaffold, indicating successful phase separation and ice crystal templating during subzero polymerization. We hypothesize that the decrease in AAD concentration led to decreased mechanical strength resulting in irregular shaped pores due to the pore walls being less strong possibly due to lower degree of covalent crosslinking. Quantitative analysis demonstrated that the 20% (*w*/*v*) PEG–alginate DN cryogels had a mean pore size of 15.03 ± 6.81 μm in the middle region and 14.28 ± 6.64 μm in the bottom region. In comparison, the 10% (*w*/*v*) cryogels exhibited mean pore sizes of 26.29 ± 11.24 μm (middle) and 16.68 ± 6.14 μm (bottom). The 5% (*w*/*v*) cryogels showed average pore sizes of 31.66 ± 13.82 μm (middle) and 13.41 ± 5.23 μm (bottom) (Figure 2), respectively, suggesting a relatively broad size distribution. The PEG–alginate DN cryogels did not show a significant change in pore size when the crosslinker was switched to EDGMA from DTT. These cryogels have a pore size of 10.38 ± 0.48 μm in the bottom section and 17.99 ± 5.70 μm in the middle section. These pore sizes are comparable to those previously reported for 20% *w*/*v* hybrid DN PEG–alginate cryogels formed via click-based and ionic crosslinking, which ranged from 4 to 40 μm [25]. Compared to DN cryogels the pore size for SN alginate was significantly larger while SN PEG (Supplementary Figure S1A) cryogels had a similar or smaller pore size (8–20 µm) with higher uniformity, indicating that combining two networks leads to a loss in porosity (Figure 2).

Large pore sizes in cryogels are also expected to increase the swelling ratio and facilitate quick equilibrium with the surrounding medium. The interconnected macroporous network is expected to facilitate efficient mass transport, and support high-density cell infiltration, making cryogels promising candidates for tissue engineering applications [14,40,41].

### 2.3. Compressive Mechanical Properties of DN Cryogels

The compression test was conducted on PEG–alginate DN cryogels with varying concentration of PEG, and the results are compiled in Table 1**.** The results showed that 20% PEG–alginate cryogels made using DTT as the crosslinker had a compressive modulus of 141.28 ± 34 kPa, 10% PEG–alginate DN cryogels exhibited an intermediate modulus of 113.33 ± 1 kPa, and 5% PEG–alginate DN cryogels had the lowest modulus, at 84.83 ± 1 kPa. Thus, as PEG concentration decreased, the mechanical strength of the cryogels decreased significantly. Interestingly, although modulus was the largest for the 20% PEG–alginate cryogels among all the cryogels containing DTT, a wider error was found for these 20% PEG–alginate cryogels due to possible network structure heterogeneity at these high concentrations. Conversely, reasonably consistent modulus was displayed by the 10% PEG-containing DN cryogels with a smaller error and improved stability.

Interestingly, in DN cryogels where the 20% PEG network was crosslinked using an alternate dithiol crosslinker, EGBMA resulted in a markedly higher compressive modulus of 481.41 ± 132 kPa, which was three times that of 20% PEG-alginate cryogels containing DTT containing. This sharp enhancement indicates the greater degree of crosslinking in EGBMA-containing DN cryogels, promoting a more rigid network. Despite the relatively large error range, the mechanical performance of the 20% PEG-containing DN cryogels crosslinked with EGBMA cryogels clearly surpasses that of the DTT-based systems, suggesting that EGBMA produced a higher compressive modulus under monotonic compression compared with the DTT-based formulation.

The variation in mechanical properties can be attributed to both the density and the type of network structure formed at different polymer concentrations, as well as to the crosslinker structure. At high PEG concentrations, dense three-dimensional networks form, yielding an elevated modulus, whereas at lower PEG concentrations the pore walls are likely to be less dense, resulting in a lower modulus. The crosslinker further modulates this behavior, as EGBMA markedly enhances the mechanical strength of the cryogels. Although high-concentration PEG cryogels could be prepared consistently, their mechanical properties varied significantly relative to low-PEG cryogels. This increased variability reflects the sensitivity of mechanical properties to composition and processing at high PEG content, where elevated precursor viscosity promotes heterogeneous mixing and heterogeneous reaction. In contrast, the 10% PEG cryogels showed greater consistency in modulus, suggesting a better balance between mechanical reinforcement and processing reproducibility.

In comparison to the ionically crosslinked PEG–alginate hybrid DN cryogels reported previously by our group [18,25], the DN cryogels developed in this study exhibit substantially improved mechanical performance. Specifically, DTT-crosslinked DN cryogels containing 20 wt% PEG display an approximately 2.6-fold increase in compressive modulus relative to the prior ionically crosslinked counterparts, indicating that covalent crosslinking of the alginate network confers superior mechanical strength. Similarly, 20 wt% PEG–alginate DN cryogels crosslinked using EGBMA exhibit a 1.2-fold increase in compressive modulus compared to previously reported hybrid DN cryogels with ionically crosslinked alginate. Importantly, these compressive moduli fall within, or approach ranges reported for selected soft tissues, which typically exhibit compressive moduli approximately in ranges of 0.1–1 MPa [3]. By crosslinking alginate with AAD, our approach enables exclusively covalent network formation, yielding macroporous scaffolds with improved compressive properties relative to single-network cryogels of either alginate or PEG alone (Table 1 and Supplementary Information Figure S1C,D). This comparison with the single-network controls further indicates that PEG is the major contributor to the mechanical strength of the DN cryogels; yet, combining the two networks produces a significant increase in compressive modulus even at the lowest PEG concentration of 5%. Collectively, these findings show that covalently crosslinked PEG–alginate DN cryogels exhibit improved compressive properties under monotonic loading and may provide a useful platform for soft tissue scaffold development.

### 2.4. Degradation Kinetics of the Cryogels

**Figure 3:**
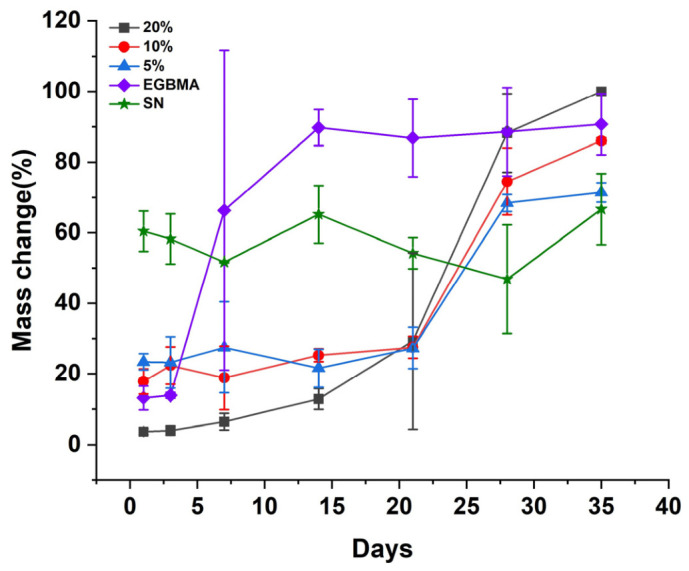
In vitro mass-loss behavior of PEG–alginate DN cryogels. Time-dependent mass-loss profiles of PEG–alginate DN cryogels prepared with different PEG concentrations, including 20%, 10%, and 5% PEG, EGBMA-crosslinked DN cryogels, and alginate–AAD single-network cryogels. Mass loss was used as a descriptive readout of degradation-associated changes and may include contributions from chemical degradation, component release, swelling-induced relaxation, and structural reorganization. Data is presented as mean ± SD (*n* ≥ 3 cryogels).

The mass loss characteristics of PEG–alginate DN cryogels under in vitro conditions were examined over a 35-day period in DMEM/F12 culture medium (Figure 3). Mass loss of the DN cryogels depended on formulation. Apparent average mass-loss rate (Figure S2) is provided in the Supporting Information.

Our results indicated that DN cryogels with varying PEG content had varying degradation patterns. Meanwhile, EGBMA DN cryogels and alginate SN cryogels exhibit significantly different degradation behaviors consistent with their network structure and chemical bond type (Figure 3). For the initial degradation phase (1–14 days), the 5% and 10% groups, experienced a mass loss of around 15–25%. The 20% group with a denser structure experienced a gradual downward trend in mass at first. During the same phase, EGBMA DN cryogels showed more pronounced early mass changes, rapidly rising to higher levels within approximately 7–14 days, suggesting that their networks were more prone to degradation in the early stages and entering an accelerated degradation phase; in contrast, SN alginate cryogels exhibited relatively gradual mass changes throughout the early phase, suggesting that their overall structure was more stable and their degradation was relatively slow. On day 21, all groups gradually converged on their level of degradation (around 27–29%), but wide variability within the 20% group meant that several samples entered a period of intense degradation. On a longer-term basis, all groups experienced a remarkable stepwise acceleration of their mass loss between 21 and 28 days. The 20% DN PEG–alginate cryogels reached nearly 90% mass loss by 28 days and 100% (full clearance) by 35 days. The 10% PEG-containing DN cryogel reached around 86% mass loss by 35 days, and the 5% group only reached around 71% mass loss. EGBMA DN cryogels reached near-terminal mass change levels at an earlier timepoint of ~12 days and remained at a high plateau in subsequent periods (Figure 3). The faster degradation at an earlier time point is consistent with our prior studies showing complete degradation of EGBMA-containing hybrid DN PEG–alginate cryogels at 10 days [25]. In contrast, SN alginate cryogels had a limited mass change over a period of 35 days, as displayed in Figure 3. This shows that without cooperative contribution of a DN and possibly the hydrolyzable bonds in the PEG network, the alginate crosslinked via AAD maintains its structure.

Collectively our data suggests that a higher content of PEG entails both acceleration and completeness of end-point degradation on a longer-term basis. This may happen because the high content of PEG forms more hydrolyzable ester linkages and thicker pores. When it reaches a critical mass, they readily induce erosion to cause total network collapse and sudden acceleration of mass loss [35]. Low-concentration PEG cryogels, on the other hand, dissolve slowly and experience a relative degree of integrity within the residual alginate backbones, resulting in temperate and incomplete rather than complete end-point degradation. Consistent with this, the faster degradation rate in the EGBMA group further supports the contribution of “crosslinked chemical bond stability” to network destabilization, which is also consistent with our earlier studies showing that hydrolytic susceptibility of the crosslinker affects the overall mass loss rate of the hydrogels [25,35]. In contrast, the SN alginate cryogel lacks the coupling structures in the DN that are more likely to trigger overall collapse. This makes it difficult for the SN alginate cryogel to achieve the step acceleration observed in the DN system.

Additionally, due to its special macroporous structure, the entry of culture medium and diffusional passage of mass loss products promotes sudden acceleration of the degradation process once a threshold level is achieved. Nonetheless, the mass loss of the entire gel is controlled by the PEG content, as well as by the chemical stability of the linkages and the porous structure. Overall, the comparison results between EGBMA and SN further indicate that, in addition to PEG content, network configuration (DN vs. SN) and crosslinking bond type jointly determine the initiation time, acceleration mode, and terminal degree of degradation.

### 2.5. Rheological Properties of DN Cryogels

**Figure 4:**
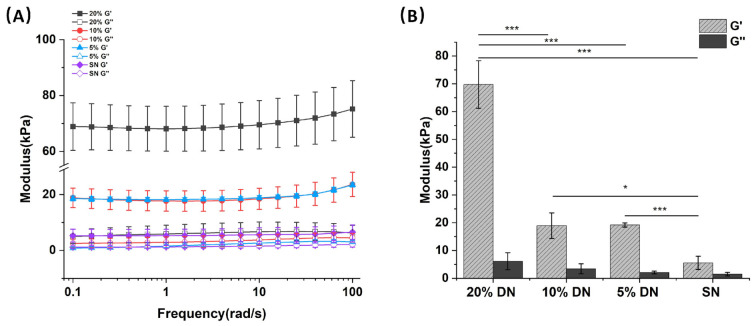
Rheological characterization of PEG–alginate double-network (DN) cryogels. (**A**) Representative storage modulus: (**A**) Representative storage modulus (G’) (filled markers) along with loss modulus (G’’) (open markers) for (box) 20%, (circle) 10%, and (triangle) 5% PEG–alginate DN cryogels, together with alginate–AAD single-network (SN) cryogels (diamond), obtained from oscillatory frequency sweep measurements. (**B**) Mean storage modulus (G′) and loss modulus (G′’) for SN cryogels and DN PEG–alginate cryogels measured at 0.1% strain over a frequency range [1–100] rad/s. * indicates *p* < 0.05, *** indicates *p* <0.001 (*n* ≥ 3).

Rheological properties of various types of cryogels in the angular frequency range [0.1–100] rad/s, including the storage modulus (G′) and loss modulus (G″) are displayed in Figure 4. The G′ values of every cryogel sample are significantly more than the corresponding G″ values, indicating that these cryogels exhibit typical solid elastic behavior in the entire frequency range and have an elastic dominant characteristic. For these studies we only studied effect of PEG concentration on the rheological properties as our previous studies show the change in dithiol crosslinker does not influence the rheological properties of the cryogels [25].

The 20% DN cryogel exhibits the highest G′ value (approximately 65–80 kPa), which is significantly higher than that of other samples, indicating that its network structure is the densest and possesses the highest mechanical strength. The G′ values of the 10% and 5% DN cryogels are approximately 18–25 kPa, and the two perform similarly; however, the 10% sample exhibits a slightly higher G′ value in the high-frequency region, indicating that its network rigidity is slightly stronger. The single-network alginate cryogel exhibits the lowest G′ and G″ values, indicating that its mechanical properties are significantly lower than those of the DN structure, confirming that the PEG network strengthens alginate network. This suggests that the introduction of the DN structure enhances the elastic modulus of the gel.

Additionally, both G′ and G″ exhibit a slight upward trend with increasing frequency, particularly in the high-frequency range. This phenomenon reflects that the network structure has a higher energy storage capacity and a slight viscous response under rapid external forces. This viscous response can be attributed to squeezing water out of the capillary network formed by interconnected pores. Resembling the cartilage whereby water flow under mechanical stress provides poroelasticity [42]. In summary, the introduction of a DN structure significantly improves the storage modulus of the cryogel, and as the proportion of the DN increases, the mechanical properties also gradually improve. Twenty percent PEG–alginate DN cryogel shows the highest elastic properties and is suitable as a candidate biomaterial in applications that require high mechanical strength.

### 2.6. Swelling Kinetics of DN PEG–Alginate Cryogels

**Figure 5:**
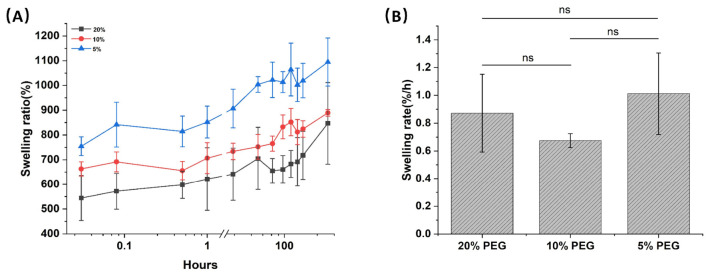
(**A**) Swelling behavior of PEG–alginate double-network (DN) cryogels prepared with different PEG concentrations. (**B**) Swelling rates of PEG–alginate DN cryogels. Analysis was statistically assessed using one-way ANOVA which preceded Tukey’s post hoc test; ns indicates no significant difference (*p* > 0.05); *n* ≥ 3 cryogels.

Figure 5 illustrates the swelling behavior of DTT crosslinked PEG–alginate DN cryogels with varying PEG content (20%, 10%, and 5%) in PBS buffer. All samples rapidly absorb water and swell in the initial stage (2 min to 1 h), after which the swelling rate slows, entering a more gradual swelling phase. Throughout the swelling process, the 5% DN cryogels had the highest swelling ratio, reaching approximately 1100% in 14 days. The 10% cryogels had the second highest swelling ratio, ultimately stabilizing at approximately 900%. The 20% cryogels had the lowest swelling ratio, reaching only approximately 850% in 14 days.

Furthermore, differences in swelling among the three groups were already evident within 1 h, and the differences between the groups widened with time. Statistical analysis revealed significant differences in swelling ratio between the 5% and 20% groups at most time points, while the differences between the 10% and 20% groups were not significant at some time points. These results indicate that as a percent of PEG content increases within the DN cryogels the swelling ratio decreases. This trend correlates with the difference in the average pore sizes of 5% and 20% PEG–alginate DN cryogel with 5% DN cryogels having a lower PEG concentration and higher pore sizes leading to a more substantial water absorption capacity and a higher final swelling ratio. Further compared to 20% PEG single-network cryogels, 20% PEG–alginate DN cryogels showed slower swelling kinetics and lower swelling ratio at comparable timepoints (Figure 5 and Supplementary Figure S1B). For instance, at 1 h the swelling ratio for 20% PEG–alginate DN was ~590 while for SN PEG cryogel it was 790 indicating a faster and higher water uptake.

Thus, overall PEG–alginate DN cryogels exhibited improved mechanical performance and distinct swelling behavior, supporting the contribution of the alginate network within the DN structure. In combination with the alginate–AAD single-network control, these results suggest that neither network alone fully recapitulates the behavior of the PEG–alginate DN cryogels. Rather, the DN formulation provides a combined-network effect that improves scaffold properties compared with the corresponding single-network systems.

### 2.7. Cell Culture in the PEG–Alginate DN Cryogels

**Figure 6:**
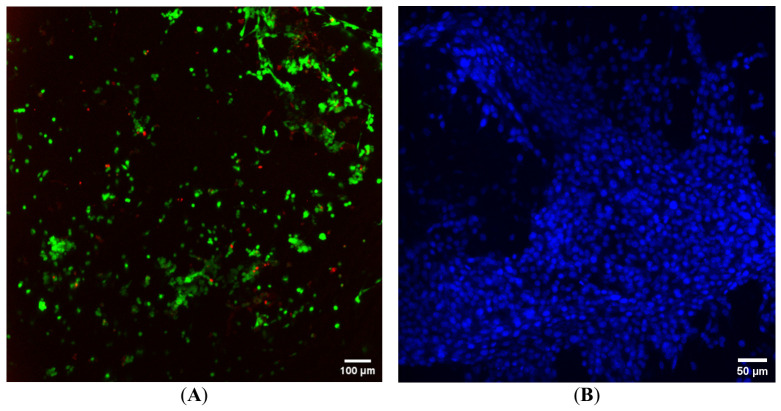
(**A**) Culture of D1 cells in PEG–alginate double-network (DN) cryogels. Live/Dead staining of D1 cells seeded in 20% PEG–alginate DN cryogels crosslinked with DTT after 7 days of culture. (**B**) DAPI staining of D1 cells seeded in DTT-crosslinked 20% PEG–alginate cryogels.

PEG–alginate DN cryogels were coated with collagen to introduce cell-adhesive sites. D1 cells were selected because their interactions with the extracellular matrix resemble those of human mesenchymal stem cells [43]. At 7 days after seeding, the viability of D1 cells within the cryogels was maintained 95.4 ± 3.8%, demonstrating excellent biocompatibility of the scaffold. In addition, the cells were able to penetrate the cryogels with high efficiency, as indicated by the cell densities measured at the bottom, middle, and top regions of the scaffold. Cell infiltration was quantified by counting the number of DAPI-stained nuclei per square millimeter. As shown in Figure 6, a maximum cell density of 1760.34 ± 914.03 cells/mm^2^ was achieved. These findings indicate that the macroporous architecture of the scaffold supports uniform and efficient cell seeding, leading to high cellular density throughout the construct.

### 2.8. Chondrogenic Differentiation of Mouse MSCs in PEG–Alginate DN Cryogels

We next evaluated the chondrogenic differentiation of mouse D1 MSCs cultured within the cryogels in conditioned media derived from macrophages in different polarization states (M0, M1, and M2). Because disease conditions such as arthritis and cartilage defects generate an inflammatory microenvironment that can drive aberrant MSC differentiation [44,45], this experiment was designed to (i) assess the ability of the cryogels to support chondrogenic differentiation under simulated inflammatory conditions, (ii) characterize MSC–macrophage paracrine interactions, and (iii) establish the potential of the cryogels as porous scaffolds supporting cell infiltration, differentiation, and paracrine signaling.

**Figure 7:**
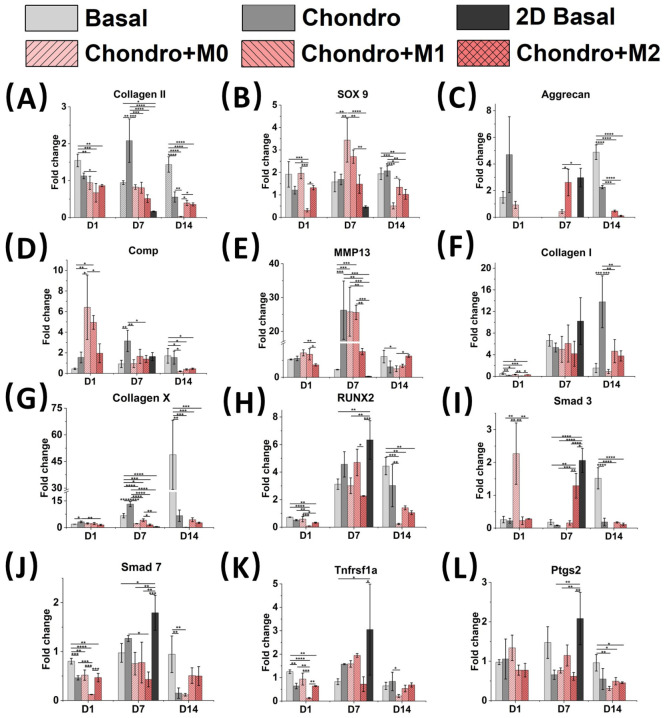
Gene expression profiles of D1 MSCs cultured in cryogels under chondrogenic conditions and supplemented with conditioned media from macrophages in different activation states. (**A**–**D**) Chondrogenic markers, including Collagen II, SOX9, Aggrecan, and COMP, were used to assess chondrogenic differentiation and cartilage matrix production. (**E**–**H**) MMP13, Collagen I, Collagen X, and RUNX2 were analyzed as markers related to matrix remodeling, fibrocartilage formation, hypertrophy, and osteogenic differentiation. (**I**,**J**) Smad3 and Smad7 were examined to evaluate TGF-β/Smad signaling activity. (**K**,**L**) Tnfrsf1a and Ptgs2 were measured as inflammation-associated genes. Data are shown as fold change with mean ± SD. All data are represented as mean ± standard deviation (*n* = 3) with statistical significance indicated as * *p* < 0.05, ** *p* < 0.01, *** *p* < 0.001, and **** *p* < 0.0001.

Overall, D1 MSCs cultured in the cryogels responded to the chondrogenic environment at early time points, although their gene expression profile was not purely hyaline cartilage-like. At Day 7, cells cultured in chondrogenic media expressed the chondrogenic markers Collagen II, SOX9, aggrecan, and COMP (Figure 7A–D). Surprisingly, SOX9 was also upregulated on Day 7 (Figure 7B), particularly in cells cultured in the M0- and M1- but to a lesser degree in M2-conditioned medium groups, suggesting that macrophage-derived factors may transiently enhance early chondrogenic transcriptional activity. This finding is consistent with reports that pro-inflammatory signaling can, under certain conditions, promote rather than suppress chondrogenesis, for example, transient TNF-α exposure has been shown to enhance MSC chondrogenic capacity and GAG deposition [46], indicating that inflammation can play a dual [47], context-dependent role in regulating chondrogenesis. However, sustained overproduction and accumulation of pro-inflammatory factors is well documented to inhibit MSC chondrogenesis, in part through NF-κB–mediated suppression of SOX9 [45]. Consistent with the recognized challenge of maintaining durable MSC chondrogenesis [48,49], we observed a decline in chondrogenic gene markers by Day 14. 

Aggrecan showed a more variable pattern, with higher expression in the chondrogenic group at early time points and a marked increase in the basal media group at Day 14 (Figure 7C). This indicates that matrix-associated gene expression was influenced not only by soluble chondrogenic factors but also by the 3D cryogel culture environment, consistent with reports that three-dimensional culture alone can sustain chondrogenic matrix gene expression in MSCs [50], and highlighting the role of the cryogel platform in supporting cellular differentiation.

Chondrogenic differentiation of MSCs is known to progress to expression of matrix remodeling and hypertrophic genes leading to fibrocartilage formation [48,49]. Thus, we also tested expression of the matrix remodeling, fibrocartilage, hypertrophy, and osteogenesis-associated genes including MMP13, Collagen I, Collagen X, and RUNX2 under various inflammatory conditions (Figure 7E–H). Our results showed that chondrogenic induction was accompanied by undesired remodeling and maturation signals. MMP13 was markedly elevated at Day 7 (Figure 7E), especially in the chondrogenic, M0-, and M1-conditioned media groups, suggesting active-matrix remodeling. Collagen I increased strongly at Day 14 in the chondrogenic group (Figure 7F). Notably, the chondrogenic conditions that supported higher SOX9 expression also showed elevated MMP13 and Collagen I, indicating a concurrent effect of macrophage- and chondrogenic-media factors on both differentiation and matrix remodeling. Collagen X and RUNX2 were likewise elevated at later time points (Figure 7G,H), pointing toward a tendency for fibrocartilaginous matrix formation, hypertrophic differentiation, or partial osteogenic commitment rather than stable hyaline cartilage. This co-induction of hypertrophic and remodeling markers (Collagen X, RUNX2, MMP13) alongside chondrogenic genes is a well-recognized limitation of TGF-β–driven MSC chondrogenesis, in which the differentiated phenotype is unstable and prone to endochondral, hypertrophy-like maturation [48,49,51].

We also tested for the TGF-β/Smad signaling-related genes including Smad3 and Smad7. TGF-β is a core driving signal for MSC chondrogenesis, primarily functioning through the TGFBR1/TGFBR2 → SMAD2/3/4 axis [52,53,54,55]. Smad3, in particular, is critical for cartilage matrix secretion and suppresses Runx2-inducible programs such as MMP13 that are detrimental to stable cartilage phenotypes [56,57]. Smad3 was transiently higher in the M0-conditioned media group at Day 1, in the M2-conditioned media on Day 7 and in the basal cryogel group at Day 14, whereas Smad7 increased mainly at Day 7, especially in the chondrogenic and 2D basal groups. This pattern suggests that TGF-β-related signaling was activated but also subjected to feedback regulation during culture, consistent with the established role of Smad7 as a TGF-β-inducible inhibitory Smad that limits SMAD2/3 activation through a negative-feedback loop [55,58]. Finally, for the inflammation-related genes Tnfrsf1a and Ptgs2, expression was generally moderate and did not show sustained inflammatory activation in the macrophage-conditioned medium groups. Both genes tended to decrease by Day 14, particularly in the M0-conditioned media group, suggesting that the conditioned media did not continuously drive a strong inflammatory response in this cryogel culture system. The generally modest inflammatory-gene expression is also consistent with the limited, transient Smad7 induction observed here, given that pro-inflammatory cytokines such as TNF-α and IL-1 can upregulate Smad7 and thereby attenuate TGF-β/SMAD2/3 signaling, a recognized point of crosstalk between inflammatory and TGF-β pathways [55,58].

These results indicate that both pro-inflammatory and anti-inflammatory environments modulate distinct signaling pathways at different time points, suggesting that a fine balance between these signals may be important for achieving sustained chondrogenic differentiation [44,45,46,59].

**Figure 8:**
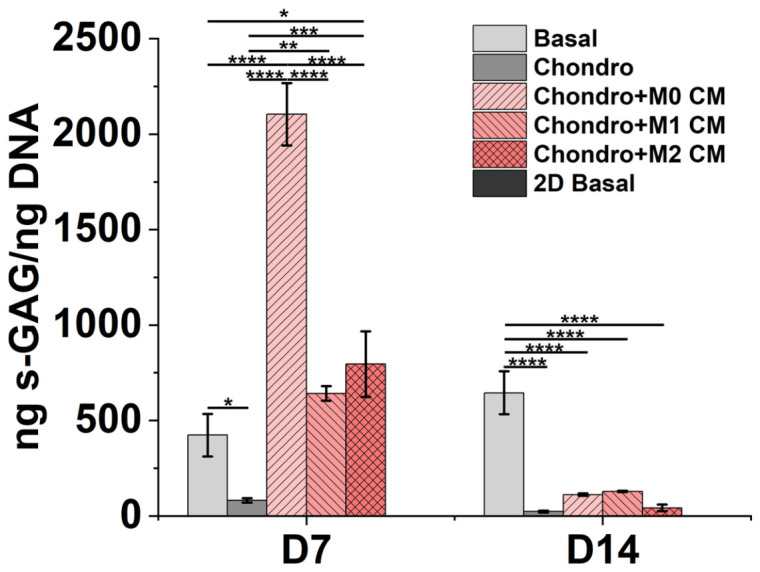
Quantification of s-GAG production by D1 MSCs cultured in PEG–alginate DN cryogels. s-GAG content was normalized to DNA content and expressed as ng s-GAG/ng DNA after 7 and 14 days of culture under different medium conditions, including basal medium, chondrogenic medium, and chondrogenic medium supplemented with M0-, M1-, or M2-conditioned medium. Data are presented as mean ± SD. Statistical significance is indicated by asterisks: * *p* < 0.05, ** *p* < 0.01, *** *p* < 0.001, and **** *p* < 0.0001.

Together, these qPCR results indicate that macrophage-conditioned medium can transiently modulate the chondrogenic activity of D1 MSCs within the cryogels, although stable cartilage-like differentiation was not achieved. To determine whether these transcriptional changes were accompanied by actual cartilage matrix production, sulfated glycosaminoglycan (s-GAG) content, a functional readout of proteoglycan-rich extracellular matrix deposition was quantified by DMMB assay and normalized to DNA content. At Day 7, M0-conditioned medium produced the greatest s-GAG secretion (~2100 ng s-GAG/ng DNA), significantly higher than all other groups. s-GAG levels in cells cultured in M1- and M2-conditioned media were also elevated relative to chondrogenic media alone, though considerably lower than in the M0 group. Notably, cells in chondrogenic media alone had the lowest s-GAG content at Day 7, indicating that chondrogenic medium by itself was insufficient to drive matrix deposition in the cryogel system, whereas the elevated deposition under macrophage-conditioned media is consistent with reports that macrophage-derived paracrine factors enhance MSC matrix production [59,60]. Cells in basal media showed moderate s-GAG secretion, suggesting that the three-dimensional cryogel environment alone supports some matrix deposition. By Day 14, s-GAG/DNA ratios decreased significantly across all macrophage-conditioned groups, most markedly in the M0 group, indicating that the stimulatory effect observed on Day 7 was no longer present. In contrast, the basal media group retained a comparatively high s-GAG/DNA ratio, again supporting role of the 3D cryogel matrix in supporting chondrogenic differentiation. This pattern shows that macrophage-conditioned medium, particularly M0-conditioned medium—induces a transient increase in s-GAG matrix formation at Day 7 that is not sustained, paralleling the transient transcriptional response seen by qPCR. Taken together, the DMMB findings indicate that matrix deposition in the cryogels is governed both by culture time and by the type of conditioned medium, reinforcing the transient, context-dependent nature of the macrophage–MSC paracrine effect in this system (Figure 8).

## 3. Conclusions

This study describes a one-pot process involving a thiol-Michael addition and carbodiimide coupling to construct PEG–alginate double-network (DN) cryogels. The PEG–alginate DN cryogels created by gelation at subzero temperatures present interconnected macropores, elastic-dominant viscoelasticity, tunable mechanical integrity, swelling, and controlled in vitro degradation. A balance between strength, stability, and speed of degradation is achievable by varying PEG content and crosslinking chemistry (DTT or EGBMA). These findings show that PEG content and crosslinker selection provide a practical strategy to tune the overall balance among stiffness, swelling, pore architecture, and degradation behavior. Degradation is jointly governed by network chemistry and crosslinker structure: high PEG promotes greater mass loss and accelerated clearance, while lower PEG yields slower hydrolysis with partial integrity retention. The durable pore walls and large channels enable rapid swelling, mass transfer, and uniform cell loading, with high cell viability maintained over 7 days. Moreover, the cryogels promote early chondrogenesis in MSCs in basal media and conditioned media from macrophages supplemented with chondrogenic media, indicating their role as support matrix. In conclusion, DN PEG–alginate cryogels hold potential as scaffolds for tissue engineering of soft tissues.

## 4. Materials and Methods

### 4.1. Materials

8-arm polyethylene glycol acrylate (8-arm PEG acrylate, 10 kDa; Jenkem Technology, Plano, TX, USA), sodium alginate with an M/G ratio of 0.6 (Protanal^®^ LF 10/60, IFF Pharma Solutions, Wilmington, DE, USA), 1-ethyl-3-(3-dimethylaminopropyl)carbodiimide (EDC; Thermo Fisher Scientific, Waltham, MA, USA), N-hydroxysuccinimide (NHS; Tokyo Chemical Industry, Tokyo, Japan), 2-(N-morpholino) ethanesulfonic acid buffer (MES, 1.0 M, pH 7.0; Thermo Fisher Scientific), and adipic acid dihydrazide (AAD; Tokyo Chemical Industry). Dithiothreitol (DTT) and ethylene glycol bis(mercaptoacetate) (EGBMA), Dulbecco’s Modified Eagle Medium/Nutrient Mixture F-12 (DMEM/F12, 1:1), fetal bovine serum (FBS), penicillin–streptomycin (PS), L-glutamine, Dulbecco’s phosphate-buffered saline (DPBS), and 0.05% trypsin–EDTA, Rat tail collagen type I, The LIVE/DEAD Viability/Cytotoxicity Kit, 4′,6-diamidino-2-phenylindole (DAPI), and 4% paraformaldehyde (PFA), ascorbic acid (Thermo Fisher Scientific). Dexamethasone (Gibco, Grand Island, NY, USA), insulin–transferrin–selenium (ITS (1X), Sigma-Aldrich, Saint Louis, MO, USA), D1 mouse mesenchymal stem cells (ATCC, passage 11).

### 4.2. PEG–Alginate Double-Network Cryogel Synthesis

This synthesis was adapted from previously reported PEG–alginate cryogel with some modifications involving crosslinking of alginate via carbodiimide-coupling methods [25,61]. The DN cryogels were produced by simultaneously crosslinking sodium alginate network and 8-arm PEG-acrylate polymeric network. Sodium alginate (2.4% *w*/*v*) was dissolved in 0.1 M MES buffer first and stirred for at least 24 h at room temperature. EDC and NHS were then slowly added under continuous stirring at a 2:1:2 (EDC:NHS:COO^-^) molar ratio. Briefly, 23 mg of EDC and 6.93 mg of NHS were added to 1 mL of a 2.4% alginate solution. Stirring continued for 30 min to ensure uniform mixing and activation of the carboxyl groups in alginate. Different concentrations of 8-arm PEG-acrylate were then incorporated into the activated alginate solution, followed by centrifugation for 5 min at 3000 rpm to eliminate air bubbles. The resulting 8-arm PEG-acrylate precursor solutions, containing 5%, 10%, and 20% (*w*/*v*), were pre-cooled on ice before use. Then, a 10× crosslinker stock solution was made by dissolving 0.8 M dithiol crosslinker (DTT or EGBMA) and 0.5 M AAD in 0.1 M MES buffer and pre-cooled briefly (≤10 min) to match the precursor temperature; extended chilling was avoided to prevent AAD precipitation. The crosslinker stock solution was added to the PEG-acrylate/alginate precursor mixture in a transparent flat-bottom tube, followed by vortexing for 15 s. The samples were then kept in a –20 °C thermostat for at least 18 h. The cryogels that resulted were thawed, washed with buffer, air-dried, and stored at room temperature until further use. For single-network (SN) cryogels, similar concentration of alginate and AAD as in DN cryogels was used, and the cryogels were made at –20 °C.

### 4.3. Scanning Electron Microscopy of Cryogels

The top, middle, and bottom microstructure portions of the cryogels were characterized via scanning electron microscopy (SEM, JEOL JSM-IT100LA, Tokyo, Japan). Prior to imaging, the cryogels were sectioned into discs with a thickness of 2–3 mm, then passed through a graded ethanol dehydration series of 20%, 40%, 60%, 80%, and 100% (*v/v*), and then samples were gold coated with a sputter coater (Desk V, Denton Vacuum, Moorestown, NJ, USA) [25]. Three micrographs were randomly selected from three independent cryogel samples for each group, with at least 100 pores measured per sample. The average pore size was quantified in ImageJ software (ImageJ version 1.54p, National Institute of Health, Bethesda, MD, USA) using thresholding and the “Measure Particles” function, and pore sizes at different locations within the cryogels were compared.

### 4.4. Swelling Kinetics of PEG–Alginate DN Cryogels

The swelling kinetics for each cryogel was assessed following the previously reported method [25]. Briefly, cryogel samples (8 mm in diameter and 5 mm in height) were dried at room temperature and the initial dry weight was recorded. Following this, the dried samples were placed in PBS. For each assigned time interval, samples were removed from the buffer, carefully blotted with Kimwipe^®^ to eliminate surface water, and weighed immediately to determine the swollen mass. Measurements were collected at 2 min, 5 min, 30 min, 1 h, and 24 h, and every 24 h following up to day 7. Afterward, the swollen mass was recorded once per week until day 14. Swelling ratio (%) was then calculated in accordance with the equation below:

Swelling Ratio % = [(M_T_ − M_G_)/(M_G_)] × 100%

M_T_ represents swollen mass, and M_G_ denotes the dry mass of the cryogel. Then using the Quickfit function in Origin, linear regression determined all swelling rates by placing swelling ratio against time.

### 4.5. Rheological Measurements of PEG–Alginate DN Cryogels

The rheological cryogel properties were assessed using a TA-DHR3 rotational rheometer (TA Instruments, New Castle, DE, USA) fitted with an 8 mm parallel-plate geometry. Cryogel samples, each with an approximate 8 mm diameter and a 6 mm height, were submerged in 1× PBS at 37 °C until acquiring equilibrium swelling, or for up to 2 h. Before measurement, the sample’s surface was wiped of excess water through gentle blotting using a KimWipe^®^ (Kimberly-Clark Professional, Roswell, GA, USA). Strain sweep experiments were performed first at a constant 1 rad/s angular frequency over a strain range [0.01–1]% to identify the viscoelastic linear region. Following this, frequency sweep tests were executed at a fixed 0.1% strain across an angular frequency range [0.1–100] rad/s. Three independent samples were analyzed for each cryogel composition, and the storage modulus (G′) and loss modulus (G″) results are presented as mean ± standard deviation.

### 4.6. Mechanical Analysis of PEG–Alginate DN Cryogels

Compression testing was performed using a Shimadzu EZ-LX universal(Shimadzu Corporation, Kyoto, Japan) testing machine (346–57300–42). Before testing, all cryogel samples were equilibrated in PBS. Cylindrical specimens with 5.75 mm of height and 8.5 mm of diameter were positioned between two flat compression plates. A 0.1 N preload force was administered to ensure full contact between the samples’ heads and the plate. The samples were then compressed with a 100 N load cell to 70% of their initial height at rate held constant at 1 mm/min, while force and displacement were recorded continuously [25].

Compressive strain (ε) was calculated as: ε = ΔL/L

ΔL represents the change in sample height, while L is the sample initial height. Compressive stress (σ) was determined according to: σ = F/A. F represents applied force while A represent the cross-sectional area. Young’s modulus (E) was obtained by calculating the slope of the linear regions of the stress–strain curve. Four samples were analyzed per group, and the mean ± standard deviation for the elastic modulus was calculated.

### 4.7. Degradation Test

All cryogels were dehydrated using a graded methanol series of 20%, 40%, 60%, 80%, and 100% (*v*/*v*). The dry weight on day 0 of each cryogel (W_0_) was recorded. Following sterilization, the cryogels were incubated in DMEM/F12 medium incorporating 1% penicillin–streptomycin and 10% fetal bovine serum (FBS) under 5% CO_2_ atmosphere at 37 °C. At predetermined incubation times, excess liquid on the sample surface was gently removed with a KimWipe^®^, and the swollen weight (Ws) was measured. At designated time points, both swollen mass (Ws) and post-incubation dry mass (Wd) were documented for all cryogels. Triplicate samples per group were utilized. The extent of degradation was calculated using the equation: Mass Change % = 100% × (W_0_ − Wd)/W_0_ [25]. Because the mass-loss profiles were non-linear, the time-dependent mass-loss curves were used as the primary readout. When reported, apparent average mass-loss rates were calculated only for descriptive comparison.

### 4.8. Collagen Coating and Cell Seeding

Murine mesenchymal stem cells (D1 line, ATCC; P11) were expanded in DMEM/F12 medium boosted with 1% penicillin–streptomycin and 10% FBS at 37 °C in a humidified 5% CO_2_ containing incubator. Once cells reached approximately 90% confluence, a 0.05% EDTA and 0.25% trypsin mix was added to separate them from the plate. The cells were then harvested and transferred to a 15 mL cylindrical centrifuge tube, then the centrifuge was run for 5 min at 1500 rpm to acquire a cell pellet. The pellet was then resuspended gently in fresh DMEM/F12 medium by pipetting, and the cells were reseeded into Petri dishes. Cryogels 1.5 mm in height and 4 mm diameter were sterilized using 70% ethanol prior to use and then incubated in a collagen type I solution (50 μg/mL) for 1 h for surface coating. Under sterile conditions, the cryogels were allowed to partially dehydrate for 2 h. Subsequently, 30 µL of cell suspension (1 × 10^5^ cells) was dispensed onto each cryogel placed in a 48-well plate. The constructs were incubated for 1 h at 37 °C to facilitate cellular infiltration and adhesion. Following this initial attachment period, each cryogel was maintained in 200 µL of complete DMEM/F12 at 37 °C in a humidified atmosphere containing 5% CO_2_, with media replaced every 48 h.

### 4.9. Cell Viability and Infiltration Measurement

To assess the biocompatibility of the cryogels, a Live/Dead assay was conducted using a commercial kit as per the instructions from the manufacturer with minor modifications. Briefly, D1 cells were cultured in the cryogels for seven days. The culture medium was then aspirated, and the cell-laden cryogel samples were washed thrice to get rid of loosely attached cells using PBS. After washing, ethidium homodimer-1 (red) and calcein-AM (green) solutions were prepared at the concentrations recommended by the manufacturer, and the samples were incubated at 37 °C for forty-five minutes in a 5% CO_2_ incubator. Afterwards, excess dye was removed through washing, and a Zeiss LSM 710 confocal microscope was used to image the samples. Utilizing ImageJ software, the ratio of total area of viable cells to that of cells which were dead was calculated.

Cryogel scaffolds were fixed in 4% paraformaldehyde (PFA) and stained with DAPI (300 nM in PBS) to evaluate cell infiltration. Like before, a Zeiss LSM 710 confocal microscope (Carl Zeiss Microscopy GmbH, Jena, Germany) was used to image the stained samples [25]. The amount of cells distributed at different depths within the cryogels was estimated with ImageJ.

### 4.10. MSC Seeding in Cryogels and Chondrogenic Differentiation

The D1 MSCs (passage 12) were cultured as described earlier. Briefly, cells were detached using trypsin-EDTA, centrifuged, and resuspended in fresh DMEM-F12 medium at the desired seeding density. Cryogel samples were prepared as previously described. A 30 μL cell suspension containing 1 × 10^5^ cells was added dropwise onto the surface of each dehydrated cryogel, followed by incubation at 37 °C for 1 h to promote cell adhesion. The cryogels were then transferred into 200 μL of complete medium and cultured at 37 °C in a humidified incubator with 5% CO_2_.

Macrophage-conditioned media was prepared using RAW 264.7 macrophages maintained as unpolarized M0 cells or polarized into M1 and M2 phenotypes. M1 polarization was induced with LPS (100 ng/mL) and IFN-γ (10 ng/mL), while M2 polarization was induced with IL-4 (10 ng/mL) and IL-13 (10 ng/mL) for 24 h. After polarization, cells were washed twice with PBS and incubated in fresh serum-free medium for another 24 h. The conditioned media was collected, centrifuged at 500× *g* for 5 min to remove cell debris and used immediately or stored at −80 °C until use.

The cryogels were divided into five groups. Group 1 consisted of cells cultured in DMEM-F12 medium supplemented with 1X ITS + 1 premix (insulin, human transferrin, and selenite), with the FBS concentration gradually reduced from 10% on day 1 to 5%, 2%, 1%, and finally 0% during subsequent medium changes. Group 2 was cultured in a chondrogenic medium consisting of DMEM-F12 with gradually reduced FBS, 20 ng/mL TGF-β1, 10 nM dexamethasone, 50 nM ascorbic acid, and 1X ITS + 1 premix. Groups 3–5 were cultured in a chondrogenic medium mixed with macrophage-conditioned M0, M1, or M2 medium, respectively, at a 2:1 ratio. The medium was changed every 48 h. As a control, cells were seeded directly onto tissue culture plastic and cultured in DMEM-F12 medium containing 10% FBS. At each time point, three samples were collected for s-GAG quantification, and gene expression analysis.

### 4.11. RNA Extraction and Target Gene Expression

Total RNA was extracted from D1 MSCs cultured on tissue culture plates and within cryogels at D1, D7, and D14 using the RNeasy Total RNA Kit (Qiagen, Valencia, CA, USA). The extracted RNA was then reverse-transcribed into cDNA using the iSCRIPT cDNA Synthesis Kit (Bio-Rad, Hercules, CA, USA). qPCR was performed using 10 ng of cDNA template and SYBR Green Master Mix on a QuantStudio 3 Real-Time PCR System (Thermo Fisher Scientific). The primers were synthesized by Thermo Fisher Scientific, with sequences obtained from previous studies and listed in Table 2 [25,62,63,64]. The target genes included SRY-box transcription factor 9(*SOX9*), Aggrecan (*ACAN*), collagen type II alpha 1(*Col2a1*), collagen type X alpha 1 (*Col10a1*), SMAD family member 3 (Smad3), SMAD family member 7 (*Smad7*), prostaglandin-endoperoxide synthase 2 (Ptgs2), tumor necrosis factor receptor superfamily member 1a (*Tnfrsf1a*), matrix metallopeptidase 13 (*MMP13*), runt-related transcription factor 2 (*Runx2*), *Collagen type 1* (*COL1*) and cartilage oligomeric matrix protein (*COMP*). Gene expression was normalized to the housekeeping gene glyceraldehyde-3-phosphate dehydrogenase (*GAPDH*). Relative expression levels were calculated using the ΔΔCt method, with D1 MSCs cultured on tissue culture plastic at day 1 used as the calibration group (Table 2).

### 4.12. Quantification of s-GAG Content by DMMB Assay

After incubation for 7 and 14 days, the cell-cryogel constructs were harvested from the gels by incubating with 200 µL of trypsin-EDTA for 5–8 min. Trypsin was then neutralized by mixing with equal volume of the complete media and the cells were centrifuged to collect a pellet. Cell pellets were thoroughly digested in papain buffer overnight at 65 °C. Sulfated glycosaminoglycan (s-GAG) content in the samples was determined using a dimethylmethylene blue (DMMB) colorimetric assay. A standard curve was established using a chondroitin sulfate standard. After reaction with DMMB reagent in a 96-well plate, the absorbance was read at 525 nm to calculate the sGAG content relative to the standards. DNA content was quantified using the PicoGreen fluorescence assay. After mixing the sample with the reagent working solution, the DNA concentration was calculated using the standard curve. The s-GAG content of each sample was normalized by its DNA content to assess the level of matrix synthesis by cells in cryogel.

### 4.13. Statistical Analysis

All quantitative results are represented as mean ± standard deviation (SD). The sample size (n) corresponds to the number of independent cryogel samples and/or biological replicates, as indicated in the figure legends. Multiple groups were compared using one-way analysis of variance (ANOVA) preceding Tukey’s post hoc tests for pairwise comparisons. *p* values < 0.05 were considered statistically significant; statistical significance was indicated as follows: * *p* < 0.05, ** *p* < 0.01, *** *p* < 0.001, and **** *p* < 0.0001.

## Figures and Tables

**Table 1:** Compressive mechanical properties of the PEG–alginate DN cryogels and alginate SN cryogel.

	Cryogel	Toughness (kJ/m^3^)	Stress at Break (kPa)	Strain at Break (%)	Compressive Modulus (kPa)
A	20% PEG with DTT crosslinker	18.10 ± 4.17	103.22 ± 15.59	42.70 ± 1.46	141.28 ± 34.17
B	10% PEG with DTT crosslinker	76.34 ± 0.67	132.11 ± 46.20	54.34 ± 6.13	113.33 ± 1.16
C	5% PEG with DTT crosslinker	64.49 ± 36.56	does not break	does not break	84.83 ± 1.07
D	20% PEG with EGBMA crosslinker	53.85 ± 2.84	112.15 ± 23.36	39.85 ± 11.52	481.41 ± 132.39
E	SN Alginate with AAD crosslinker	1.14 ± 0.35	does not break	does not break	2.34 ± 1.14

Note: (Toughness: A, B *, A, C *, B, E **, C, E **, D, E *. Strain at break: A, B *. Compressive modulus: A, D ***, B, D ***, C, D ****, D, E ****) Statistical significance is indicated by asterisks: * *p* < 0.05, ** *p* < 0.01, *** *p* < 0.001, and **** *p* < 0.0001.

**Table 2:** Primer sequences used for gene expression analysis.

Gene Name	Forward Primer Sequence	Reverse Primer Sequence
*SOX 9*	CCTTCAACCTTCCTCACTACAGC	GGTGGAGTAGAGCCCTGAGC
*ACAN*	CGCCACTTTCATGACCGAGA	TCATTCAGACCGATCCACTGGTAG
*Col2a1*	CCTCCGTCTACTGTCCACTGA	ATTGGAGCCCTGGATGAGCA
*Col10a1*	GCCAAGCAGTCATGCCTGAT	GACACGGGCATACCTGTTACC
*Smad3*	TGACAAGGTCCTCACCCAGA	CAGGCTGGTGCCTTAGTTGA
*Smad7*	GGCTGTGTTGCTGTGAATC	GGTATCTGGGAGTAAGGAGGAG
*Ptgs2*	TGAGCAACTATTCCAAACCAGC	GCACGTAGTCTTCGATCACTATC
*Collagen type 1*	TCAGAGGCGAAGGCAACAGTC	GCAGGCGGGAGGTCTTGG
*MMP13*	GATGACCTGTCTGAGGAAGACC	GCATTTCTCGGAGCCTGTCAAC
*Tnfrsf1a*	GTGTGGCTGTAAGGAGAACCAG	CACACGGTGTTCTGAGTCTCCT
*COMP*	GTGCCCAACTTTGACCAGAGTG	ACAGGCATCACCCACAAAGTCG
*GAPDH*	TGAAGCAGGCATCTGAGGG	CGAAGGTGGAAGAGTGGGAG
*RUN X2*	CCTGAACTCTGCACCAAGTCCT	TCATCTGGCTCAGATAGGAGGG

## Data Availability

The raw data supporting the conclusions of this article will be made available by the authors on request.

## Supplementary Materials

The following supporting information can be downloaded at: https://www.mdpi.com/article/10.3390/gels12060533/s1, Figure S1: Characterization of PEG single-network cryogel controls. Representative (A) SEM image, (B) Swelling behavior, (C) Representative compressive stress–strain curve and (D) Compressive modulus of PEG single-network cryogels. These data were included to clarify the contribution of the dual-network architecture. The comparison indicates that PEG–alginate DN cryogels exhibit improved mechanical performance and distinct swelling behavior relative to PEG single-network controls; Figure S2: Apparent average mass-loss rate calculated for descriptive comparison only. Because the mass-loss profiles were non-linear, this value was not used as a kinetic model of degradation. (Statistical analysis was conducted using one-way ANOVA followed by Tukey’s post hoc test. * indicates *p* < 0.05, ** indicates *p* < 0.01, *** indicates *p* < 0.001, **** indicates *p* < 0.0001 (*n* ≥ 3 cryo-gels).

## References

[1] Ho J., Walsh C., Yue D., Dardik A., Cheema U. (2017). Current advancements and strategies in tissue engineering for wound healing: A comprehensive review. Adv. Wound Care..

[2] Muzzio N., Moya S., Romero G. (2021). Multifunctional scaffolds and synergistic strategies in tissue engineering and regenerative medicine. Pharmaceutics.

[3] Guo J.-B., Liang T., Che Y.-J., Yang H.-L., Luo Z.-P. (2020). Structure and mechanical properties of high-weight-bearing and low-weight-bearing areas of hip cartilage at the micro-and nano-levels. BMC Musculoskelet. Disord..

[4] Jiang S., Wang M., He J. (2021). A review of biomimetic scaffolds for bone regeneration: Toward a cell-free strategy. Bioeng. Transl. Med..

[5] Gomez-Florit M., Pardo A., Domingues R.M., Graça A.L., Babo P.S., Reis R.L., Gomes M.E. (2020). Natural-based hydrogels for tissue engineering applications. Molecules.

[6] Taghipour Y.D., Hokmabad V.R., Del Bakhshayesh A.R., Asadi N., Salehi R., Nasrabadi H.T. (2020). The application of hydrogels based on natural polymers for tissue engineering. Curr. Med. Chem..

[7] Lee J.-H., Kim H.-W. (2018). Emerging properties of hydrogels in tissue engineering. J. Tissue Eng..

[8] Li X., Gong J.P. (2024). Design principles for strong and tough hydrogels. Nat. Rev. Mater..

[9] Zhang Y.S., Khademhosseini A. (2017). Advances in engineering hydrogels. Sci. Adv..

[10] Xu X., Jerca V.V., Hoogenboom R. (2021). Bioinspired double network hydrogels: From covalent double network hydrogels via hybrid double network hydrogels to physical double network hydrogels. Mater. Horiz..

[11] Gong J.P. (2010). Why are double network hydrogels so tough?. Soft Matter.

[12] Kharkar P.M., Kiick K.L., Kloxin A.M. (2013). Designing degradable hydrogels for orthogonal control of cell microenvironments. Chem. Soc. Rev..

[13] Li Y., Maciel D., Rodrigues J., Shi X., Tomás H. (2015). Biodegradable polymer nanogels for drug/nucleic acid delivery. Chem. Rev..

[14] Jain E., Zhang K., Mishra Tiwari R. (2025). Properties and Characterization of cryogels: Structural, mechanical, and functional insights. ACS Omega.

[15] Lozinsky V.I. (2002). Cryogels on the basis of natural and synthetic polymers: Preparation, properties and application. Russ. Chem. Rev..

[16] Okay O., Lozinsky V.I. (2014). Synthesis and structure–property relationships of cryogels. Polymeric Cryogels: Macroporous Gels with Remarkable Properties.

[17] Holkar K., Zhang K., Mishra Tiwari R., Jain E. (2025). Emerging trends in cryogelation: Key factors influencing cryotropic gelation processes. J. Polym. Sci. Eng..

[18] Yang Z., Zhang K., Seitz M.P., Jain E. (2025). Macroporous Alginate–PEG Hybrid Double Network Cryogels: Tuning Mechanics, Porosity, and Long-Term Growth Factor Release via Polymer Concentration, Ice Nucleation, and Sulfation. ACS Appl. Bio Mater..

[19] Henderson T.M., Ladewig K., Haylock D.N., McLean K.M., O’Connor A.J. (2013). Cryogels for biomedical applications. J. Mater. Chem. B..

[20] Gun’ko V.M., Savina I.N., Mikhalovsky S.V. (2013). Cryogels: Morphological, structural and adsorption characterisation. Adv. Colloid Interface Sci..

[21] Kothale D., Verma U., Dewangan N., Jana P., Jain A., Jain D. (2020). Alginate as promising natural polymer for pharmaceutical, food, and biomedical applications. Curr. Drug Deliv..

[22] Szekalska M., Puciłowska A., Szymańska E., Ciosek P., Winnicka K. (2016). Alginate: Current use and future perspectives in pharmaceutical and biomedical applications. Int. J. Polym. Sci..

[23] Savina I.N., Zoughaib M., Yergeshov A.A. (2021). Design and assessment of biodegradable macroporous cryogels as advanced tissue engineering and drug carrying materials. Gels.

[24] Sezen S., Thakur V.K., Ozmen M.M. (2021). Highly effective covalently crosslinked composite alginate cryogels for cationic dye removal. Gels.

[25] Zhang K., Yang Z., Seitz M.P., Jain E. (2024). Macroporous PEG-alginate hybrid double-network cryogels with tunable degradation rates prepared via radical-free cross-linking for cartilage tissue engineering. ACS Appl. Bio Mater..

[26] Kahveci M.U., Beyazkilic Z., Yagci Y. (2010). Polyacrylamide cryogels by photoinitiated free radical polymerization. J. Polym. Sci. A Polym. Chem..

[27] Shakya A.K., Holmdahl R., Nandakumar K.S., Kumar A. (2014). Polymeric cryogels are biocompatible, and their biodegradation is independent of oxidative radicals. J. Biomed. Mater. Res. A.

[28] Carmagnola I., Ranzato E., Chiono V. (2018). Scaffold functionalization to support a tissue biocompatibility. Functional 3D Tissue Engineering Scaffolds.

[29] Kong H.J., Kaigler D., Kim K., Mooney D.J. (2004). Controlling rigidity and degradation of alginate hydrogels via molecular weight distribution. Biomacromolecules.

[30] Lee K.Y., Rowley J.A., Eiselt P., Moy E.M., Bouhadir K.H., Mooney D.J. (2000). Controlling mechanical and swelling properties of alginate hydrogels independently by cross-linker type and cross-linking density. Macromolecules.

[31] D’souza A.A., Shegokar R. (2016). Polyethylene glycol (PEG): A versatile polymer for pharmaceutical applications. Expert Opin. Drug Deliv..

[32] Dispinar T., Van Camp W., De Cock L.J., De Geest B.G., Du Prez F.E. (2012). Redox-responsive degradable PEG cryogels as potential cell scaffolds in tissue engineering. Macromol. Biosci..

[33] Ifkovits J.L., Burdick J.A. (2007). Photopolymerizable and degradable biomaterials for tissue engineering applications. Tissue Eng. Part A.

[34] Lee K.Y., Bouhadir K.H., Mooney D.J. (2004). Controlled degradation of hydrogels using multi-functional cross-linking molecules. Biomaterials.

[35] Jain E., Hill L., Canning E., Sell S.A., Zustiak S.P. (2017). Control of gelation, degradation and physical properties of polyethylene glycol hydrogels through the chemical and physical identity of the crosslinker. J. Mater. Chem. B.

[36] Li B., Yang Z., Li Y., Zhang J., Li C., Lv N. (2024). Exploration beyond osteoarthritis: The association and mechanism of its related comorbidities. Front. Endocrinol..

[37] Yang Y., Zhang H. (2024). Intra-articular injection of nanomaterials for the treatment of osteoarthritis: From lubrication function restoration to cell and gene therapy. Adv. Funct. Mater..

[38] Zhou H., Zhang Z., Mu Y., Yao H., Zhang Y., Wang D.-A. (2024). Harnessing nanomedicine for cartilage repair: Design considerations and recent advances in biomaterials. ACS Nano.

[39] Zhu R., Schutyser M.A., Boom R.M., Zhang L. (2024). Tuning the mechanical properties of starch-based cryogels with the aid of additives. Carbohydr. Polym. Technol. Appl..

[40] Hixon K.R., Jain E., Kadakia P.U., Minden-Birkenmaier B.A., Sell S.A. (2015). Cryogel Scaffolds: An Appropriate Scaffold Structure for Promoting Bone Regeneration. Tissue Eng. Part A.

[41] Jain E., Kumar A. (2013). Disposable polymeric cryogel bioreactor matrix for therapeutic protein production. Nat. Protoc..

[42] Greene G.W., Zappone B., Söderman O., Topgaard D., Rata G., Zeng H., Israelachvili J.N. (2010). Anisotropic dynamic changes in the pore network structure, fluid diffusion and fluid flow in articular cartilage under compression. Biomaterials.

[43] Neal S., Tan X., Jain E., Chen C., Hashemi M., Setton L.A., Huebsch N. (2025). Enhancing the Potency of Growth Factor-Mimicking Peptides via Cross-Presentation with Integrin Ligands. J. Biomed. Mater. Res. Part A.

[44] Goldring M.B., Otero M., Plumb D.A., Dragomir C., Favero M., El Hachem K., Hashimoto K., Roach H.I., Olivotto E., Borzi R.M. (2011). Roles of inflammatory and anabolic cytokines in cartilage metabolism: Signals and multiple effectors converge upon MMP-13 regulation in osteoarthritis. Eur. Cells Mater..

[45] Wehling N., Palmer G.D., Pilapil C., Liu F., Wells J.W., Muller P.E., Evans C.H., Porter R.M. (2009). Interleukin-1β and tumor necrosis factor alpha inhibit chondrogenesis by human mesenchymal stem cells through NF-κB-dependent pathways. Arthritis Rheum..

[46] Voskamp C., Koevoet W., Somoza R.A., Caplan A.I., Lefebvre V., van Osch G., Narcisi R. (2020). Enhanced Chondrogenic Capacity of Mesenchymal Stem Cells After TNFalpha Pre-treatment. Front. Bioeng. Biotechnol..

[47] Kondo M., Yamaoka K., Sakata K., Sonomoto K., Lin L., Nakano K., Tanaka Y. (2015). Contribution of the Interleukin-6/STAT-3 Signaling Pathway to Chondrogenic Differentiation of Human Mesenchymal Stem Cells. Arthritis Rheum..

[48] Melnik S., Gabler J., Dreher S.I., Hecht N., Hofmann N., Grossner T., Richter W. (2020). MiR-218 affects hypertrophic differentiation of human mesenchymal stromal cells during chondrogenesis via targeting RUNX2, MEF2C, and COL10A1. Stem Cell Res. Ther..

[49] Somoza R.A., Welter J.F., Correa D., Caplan A.I. (2014). Chondrogenic differentiation of mesenchymal stem cells: Challenges and unfulfilled expectations. Tissue Eng. Part B Rev..

[50] de Vries-van Melle M.L., Narcisi R., Kops N., Koevoet W.J., Bos P.K., Murphy J.M., Verhaar J.A., van der Kraan P.M., van Osch G.J. (2014). Chondrogenesis of mesenchymal stem cells in an osteochondral environment is mediated by the subchondral bone. Tissue Eng. Part A.

[51] Zhong L., Huang X., Karperien M., Post J.N. (2015). The Regulatory Role of Signaling Crosstalk in Hypertrophy of MSCs and Human Articular Chondrocytes. Int. J. Mol. Sci..

[52] Wang W., Rigueur D., Lyons K.M. (2014). TGFβ signaling in cartilage development and maintenance. Birth Defects Res. Part C Embryo Today Rev..

[53] Li T.F., O’Keefe R.J., Chen D. (2005). TGF-β signaling in chondrocytes. Front. Biosci..

[54] Chen Z., Zhou T., Luo H., Wang Z., Wang Q., Shi R., Li Z., Pang R., Tan H. (2024). HWJMSC-EVs promote cartilage regeneration and repair via the ITGB1/TGF-beta/Smad2/3 axis mediated by microfractures. J. Nanobiotechnol..

[55] van der Kraan P.M. (2017). The changing role of TGFbeta in healthy, ageing and osteoarthritic joints. Nat. Rev. Rheumatol..

[56] Chen C.G., Thuillier D., Chin E.N., Alliston T. (2012). Chondrocyte-intrinsic Smad3 represses Runx2-inducible matrix metalloproteinase 13 expression to maintain articular cartilage and prevent osteoarthritis. Arthritis Rheum..

[57] Yang X., Chen L., Xu X., Li C., Huang C., Deng C.X. (2001). TGF-β/Smad3 signals repress chondrocyte hypertrophic differentiation and are required for maintaining articular cartilage. J. Cell Biol..

[58] Yan X., Liu Z., Chen Y. (2009). Regulation of TGF-beta signaling by Smad7. Acta Biochim. Biophys. Sin..

[59] Miyamoto Y., Kubota K., Asawa Y., Hoshi K., Hikita A. (2021). M1-like macrophage contributes to chondrogenesis in vitro. Sci. Rep..

[60] Sesia S.B., Duhr R., Medeiros da Cunha C., Wolf F., Padovan E., Spagnoli G.C., Martin I., Barbero A. (2014). M2-macrophages modulate the cartilage-forming capacity of human bone marrow-derived mesenchymal stem/progenitor cell. Osteoarthr. Cartil..

[61] Khunmanee S., Jeong Y., Park H.J. (2017). Crosslinking method of hyaluronic-based hydrogel for biomedical applications. J. Tissue Eng..

[62] Limraksasin P., Kosaka Y., Zhang M., Horie N., Kondo T., Okawa H., Yamada M., Egusa H. (2020). Shaking culture enhances chondrogenic differentiation of mouse induced pluripotent stem cell constructs. Sci. Rep..

[63] Glennon-Alty L., Williams R., Dixon S., Murray P. (2013). Induction of mesenchymal stem cell chondrogenesis by polyacrylate substrates. Acta Biomater..

[64] Namachivayam K., Blanco C.L., MohanKumar K., Jagadeeswaran R., Vasquez M., McGill-Vargas L., Garzon S.A., Jain S.K., Gill R.K., Freitag N.E. (2013). Smad7 inhibits autocrine expression of TGF-β2 in intestinal epithelial cells in baboon necrotizing enterocolitis. Am. J. Physiol. Gastrointest. Liver Physiol..

