# Supplementary material for: Ice Templated PEG–Alginate Double-Network Cryogels with Tunable Mechanics and Degradation for Soft Tissue Engineering
Source: Gels. 2026 Jun 13;12(6):533. doi: 10.3390/gels12060533 (PMC13299618; doi:10.3390/gels12060533)
Supplement: Supplementary file 1 [file gels-12-00533-s001.zip › gels-4240145-supplementary.pdf]

Ice Templated PEG–Alginate Double Network Cryogels with Tunable Mechanics and Degradation for Soft Tissue Engineering

Kaixiang Zhang [1,2] Michael Patrick Seitz [1,2], Matthew Pinto [1,†], William Ofori-Atta Eghan [1,2] and Era Jain, PhD [1,2,*]

[1]Department of Biomedical and Chemical Engineering;
[2]Bioinspired Syracuse: Institute for Material and Living System, Syracuse University,

Syracuse, NY 13244, USA

*Correspondence: Era Jain (Ph.D.),
Biomedical and Chemical Engineering,Bioinspired Syracuse: Institute for Material and Living System,
Syracuse University, Syracuse, NY, USA, 13244,
Tel: 315.443.4050,
Email: erjain@syr.edu

[†] Present Address: Department of Biocomputational Engineering at the Universities at Shady Grove in Rockville, MD.

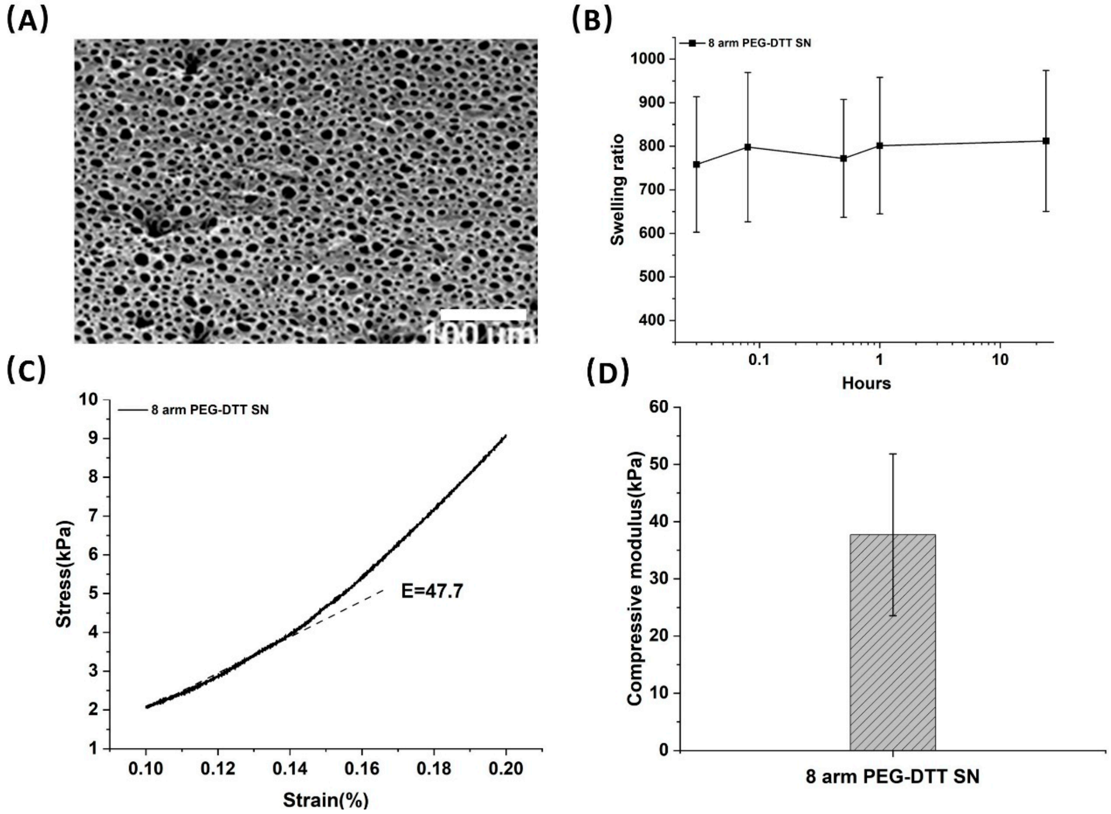

Figure S1. Characterization of PEG single-network cryogel controls.

Representative **(A)** SEM image, **(B)**Swelling behavior, **(C)** Representative compressive stress–strain curve and **(D)** Compressive modulus of PEG single-network cryogels. These data were included to clarify the contribution of the dual-network architecture. The comparison indicates that PEG–alginate DN cryogels exhibit improved mechanical performance and distinct swelling behavior relative to PEG single-network controls.

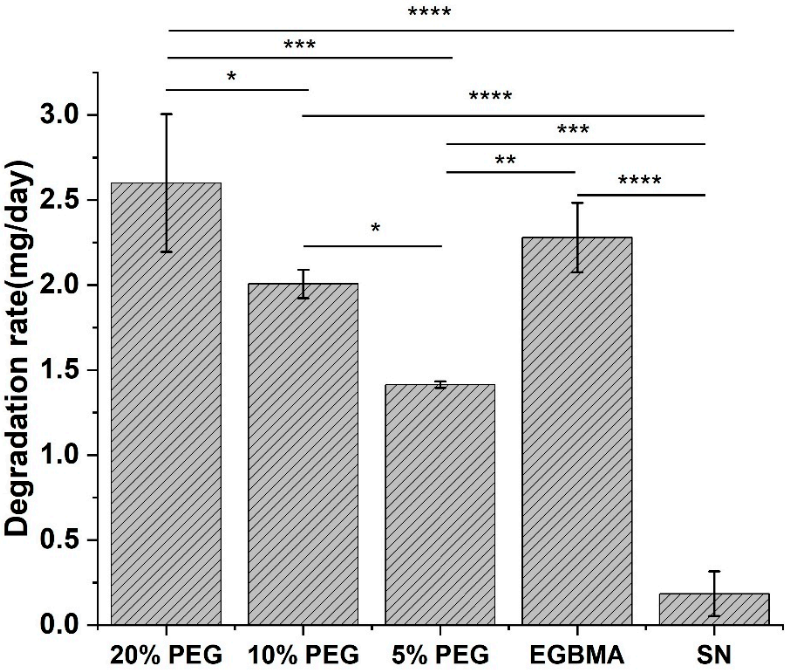

Figure S2. Apparent average mass-loss rate calculated for descriptive comparison only. Because the mass-loss profiles were non-linear, this value was not used as a kinetic model of degradation. (Statistical analysis was conducted using one-way ANOVA followed by Tukey's post hoc test. * indicates $p < 0.05$, ** indicates $p< 0.01$, *** indicates $p < 0.001$, **** indicates $p < 0.0001$ ($n \geq 3$ cryo-gels).
